# Draft Genome Sequence of the Novel Coastal Bacterium LSUCC0115 from the MWH-UniPo Clade, Order *Burkholderiales*, Class *Betaproteobacteria*

**DOI:** 10.1128/MRA.01492-19

**Published:** 2020-01-09

**Authors:** Michael W. Henson, Megan E. Guidry, M. Katherine Carnes, J. Cameron Thrash

**Affiliations:** a Department of Biological Sciences, University of Southern California, Los Angeles, California, USA; b Department of Biological Sciences, Louisiana State University, Baton Rouge, Louisiana, USA; Georgia Institute of Technology

## Abstract

Here, we present the draft genome of LSUCC0115, a novel coastal Gulf of Mexico bacterioplankton isolate within the order Burkholderiales. LSUCC0115 has the metabolic potential for aerobic heterotrophy, phototrophy, and lithoautotrophy, as well as genes for flagellar assembly and quorum sensing.

## ANNOUNCEMENT

The order *Burkholderiales* contains numerous important and numerically abundant freshwater taxa, including Polynucleobacter, Limnohabitans, and Limnobacter. We isolated an unknown betaproteobacterium designated strain LSUCC0115 from Freshwater City, LA, using high-throughput cultivation with an artificial seawater medium (1). At the time of collection, the source water had a temperature of 22.9°C and a salinity of 5.4 (1). As part of our screening process, we sequenced the 16S rRNA gene of strain LSUCC0115 (GenBank accession number KU382374) (1). A default BLASTn search of the NCBI nucleotide (nt) database found that the closest matching cultivar (outside the Louisiana State University Culture Collection [LSUCC]) was an unclassified Betaproteobacteria strain in the MWH-UniPo clade of *Burkholderiales* (94.94% identity; GenBank accession number AJ565422), isolated from eutrophic ponds in Tanzania (2). In the Gulf of Mexico, strain LSUCC0115 was the fifth most abundant operational taxonomic unit (OTU) from Freshwater City at the time of isolation (1). Due to its abundance in coastal water and its phylogenetic novelty, we chose strain LSUCC0115 for genomic sequencing and preliminary physiological analysis.

Cells for genome sequencing and temperature optimum experiments were revived from cryostocks of the Thrash Lab culture collection in sterile polycarbonate flasks containing 50 ml of JW4 medium (1). DNA for genome sequencing was extracted from a single flask after filtration using a MoBio PowerWater kit according to the manufacturer’s instructions. Library preparation and sequencing were completed at the Argonne National Laboratory Environmental Sample Preparation and Sequencing Facility as described previously (3, 4). Briefly, libraries were prepared with the PrepX ILMN kit (according to the manufacturer’s instructions) and an Apollo324 system (Mountain View, CA), size selected with the Sage Science BluePippin system (Beverly, MA), and sequenced with Illumina MiSeq technology, generating 818,414 raw paired-end (PE) 250-bp reads. Reads were quality screened and assembled with the A5-miseq pipeline (v. 20150522) using default settings (5, 6). Contamination and quality were assessed using CheckM v1.0.3 with default settings (7). The assembled genome was annotated through the NCBI Prokaryotic Genome Annotation Pipeline. Preliminary metabolic reconstruction was completed using GhostKOALA v2.2. with default settings (8, 9). The draft genome of LSUCC0115 is 1,835,506 bp in 16 scaffolds (*N*_50_, 600,205 bp), with a GC content of 59.15%, a coding density of 95.49%, and 105× coverage. The draft genome is estimated to be 98.78% complete with 0% contamination. There are 1,823 predicted genes (1,766 protein-coding genes), 44 tRNA genes, and 1 each of the 5S, 16S, and 23S rRNA genes.

**FIG 1:**
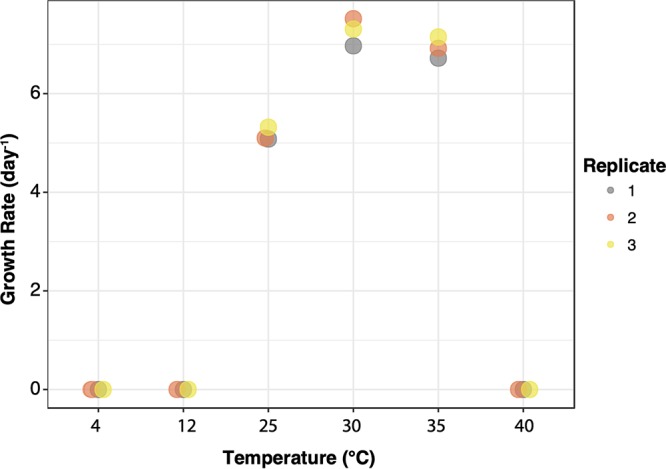
Growth rates (day^−1^) of LSUCC0115 in JW4 medium at six temperatures.

Metabolic reconstruction of LSUCC0115 suggests considerable flexibility, with the possibility for aerobic heterotrophic, lithoautotrophic, and phototrophic lifestyles. Genes are present for high- and low-affinity cytochrome oxidases, the pentose phosphate shunt, gluconeogenesis, and the tricarboxylic acid (TCA) cycle. Genes coding for potential phototrophy and lithoautotrophy included those encoding ribulose-1,5-bisphosphate; bacteriochlorophyll and carotenoid biosynthesis; the *puhA* photosynthetic reaction center and the *pufA* light-harvesting complex genes; and those encoding a complete sulfur oxidation (SOX) pathway. Temperature optimum experiments with LSUCC0115 were conducted in JW4 medium in triplicate. Growth rates were calculated as previously reported (10). LSUCC0115 grew between 25 and 35°C, with a maximum average growth rate at 30°C of 7.3 day^−1^ (Fig. 1).

### Data availability.

Sample information, genomic assembly and annotation, and raw sequences are accessible using the NCBI BioProject, whole-genome shotgun (WGS), and SRA accession numbers PRJNA579192, WJBW00000000, and SRX7052215, respectively. The genome is also available in IMG (taxon identification 2639762505). Cryostocks and/or live cultures of LSUCC0115 are available upon request.

## References

[1] Henson MW, Pitre DM, Weckhorst JL, Lanclos VC, Webber AT, Thrash JC 2016 Artificial seawater media facilitate cultivating members of the microbial majority from the Gulf of Mexico. mSphere 1:e00028-16. doi:10.1128/mSphere.00028-16. PMC4894692 27303734

[2] Hahn MW, Stadler P, Wu QL, Pöckl M 2004 The filtration–acclimatization method for isolation of an important fraction of the not readily cultivable bacteria. J Microbiol Methods 57:379–390. doi:10.1016/j.mimet.2004.02.004. 15134885

[3] Coelho JT, Henson MW, Thrash JC, Coelho JT, Henson MW, Thrash JC 2019 Draft genome sequence of strain LSUCC0112, a Gulf of Mexico representative of the oligotrophic marine *Gammaproteobacteria*. Microbiol Resour Announc 8:e00521-19. doi:10.1128/MRA.00521-19. 31270194 PMC6606908

[4] Lanclos VC, Henson MW, Doiron C, Thrash JC, Lanclos VC, Henson MW, Doiron C, Thrash JC 2019 Draft genome sequence of strain LSUCC0057, a member of the SAR92 clade of *Gammaproteobacteria*. Microbiol Resour Announc 8:e00599-19. doi:10.1128/MRA.00599-19. 31221657 PMC6588378

[5] Tritt A, Eisen JA, Facciotti MT, Darling AE 2012 An integrated pipeline for *de novo* assembly of microbial genomes. PLoS One 7:e42304. doi:10.1371/journal.pone.0042304. 23028432 PMC3441570

[6] Coil D, Jospin G, Darling AE 2015 A5-miseq: an updated pipeline to assemble microbial genomes from Illumina MiSeq data. Bioinformatics 31:587–589. doi:10.1093/bioinformatics/btu661. 25338718

[7] Parks DH, Imelfort M, Skennerton CT, Hugenholtz P, Tyson GW 2015 CheckM: assessing the quality of microbial genomes recovered from isolates, single cells, and metagenomes. Genome Res 25:1043–1055. doi:10.1101/gr.186072.114. 25977477 PMC4484387

[8] Kanehisa M, Sato Y, Morishima K 2016 BlastKOALA and GhostKOALA: KEGG tools for functional characterization of genome and metagenome sequences. J Mol Biol 428:726–731. doi:10.1016/j.jmb.2015.11.006. 26585406

[9] Tatusova T, DiCuccio M, Badretdin A, Chetvernin V, Nawrocki EP, Zaslavsky L, Lomsadze A, Pruitt KD, Borodovsky M, Ostell J 2016 NCBI Prokaryotic Genome Annotation Pipeline. Nucleic Acids Res 44:6614–6624. doi:10.1093/nar/gkw569. 27342282 PMC5001611

[10] Henson MW, Lanclos VC, Faircloth BC, Thrash JC 2018 Cultivation and genomics of the first freshwater SAR11 (LD12) isolate. ISME J 12:1846–1860. doi:10.1038/s41396-018-0092-2. 29599519 PMC6018831

